# Molecular-scale Insights into Cooperativity Switching of *x*TAB Adsorption on Gold Nanoparticles

**DOI:** 10.1021/acscentsci.3c01075

**Published:** 2024-01-04

**Authors:** Lang Xu, Rong Ye, Manos Mavrikakis, Peng Chen

**Affiliations:** † Department of Chemical and Biological Engineering, University of Wisconsin-Madison, Madison, Wisconsin 53706, United States; ‡ Department of Chemistry and Chemical Biology, Cornell University, Ithaca, New York 14853, United States

## Abstract

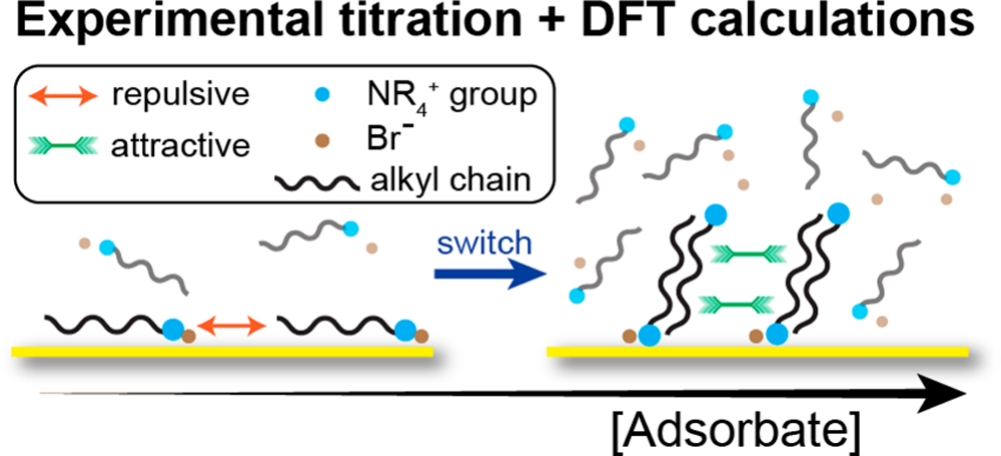

Quantifying adsorption behaviors is crucial for various applications such as catalysis, separation, and sensing, yet it is generally challenging to access in solution. Here, we report a combined experimental and computational study of the adsorption behaviors of alkyl-trimethylammonium bromides (*x*TAB), a class of ligands important for colloidal nanoparticle stabilization and shape control, with various alkyl chain lengths *x* on Au nanoparticles. We use density functional theory (DFT) to calculate *x*TAB binding energies on Au{111} and Au{110} surfaces with standing-up and lying-down configurations, which provides insights into the adsorption affinity and cooperativity differences of *x*TAB on these two facets. We demonstrate the key role of van der Waals interactions in determining the *x*TAB adsorption behavior. These computational results predict and explain the experimental discovery of *x*TAB’s adsorption behavior switch from stronger affinity, negative cooperativity to weaker affinity, positive cooperativity when the concentration of *x*TAB increases in solution. We also show that in the standing-up configuration, bilayer adsorption may occur on both facets, which can lead to different differential binding energies and consequently adsorption crossover between the two facets when the ligand concentration increases. Our combined experimental and computational approaches demonstrate a paradigm for gaining molecular-scale insights into adsorbate–surface interactions.

## Abstract 2

Combined experiments and theory reveal negative to positive cooperativity switch of ligand adsorption from lying-down to standing-up adsorption configurations on gold particles.

## Introduction

Adsorption, a process whereby a molecule becomes immobilized at an interface between two phases without being dissolved in either phase, is crucial in numerous applications, including catalysis, separation, and sensing.^1−4^ In colloidal nanoparticle chemistry, adsorption of various ligands has been widely used to control the shapes of nanocrystals during synthesis, stabilize their morphology in solution, or furnish additional surface functionality for conjugation.^5^ In these processes, the adsorption affinity of the ligands, especially their ability to differentiate different surface facets, plays a key role. Consequently, quantifying adsorption behaviors is of fundamental importance, for which many methods have been developed, such as the Brunauer–Emmett–Teller (BET) method, calorimetry,^6^ surface plasmon resonance, and spectroscopy, each with advantages and limitations depending on the nature of the adsorbate and the adsorbing surfaces.^1−4,7−15^ Theoretical methods, such as density functional theory (DFT) calculations, have been widely adopted to study adsorption on solid surfaces, particularly transition metals.^16,17^ Still, it is generally challenging to probe quantitatively the adsorption behaviors of molecules, especially in solution and on surfaces that present surface heterogeneities across different length scales (e.g., nanoparticle surfaces), for which high sensitivity and high spatial resolution methods are desired to deconvolute adsorption differences among different surface sites.

**Figure 1:**
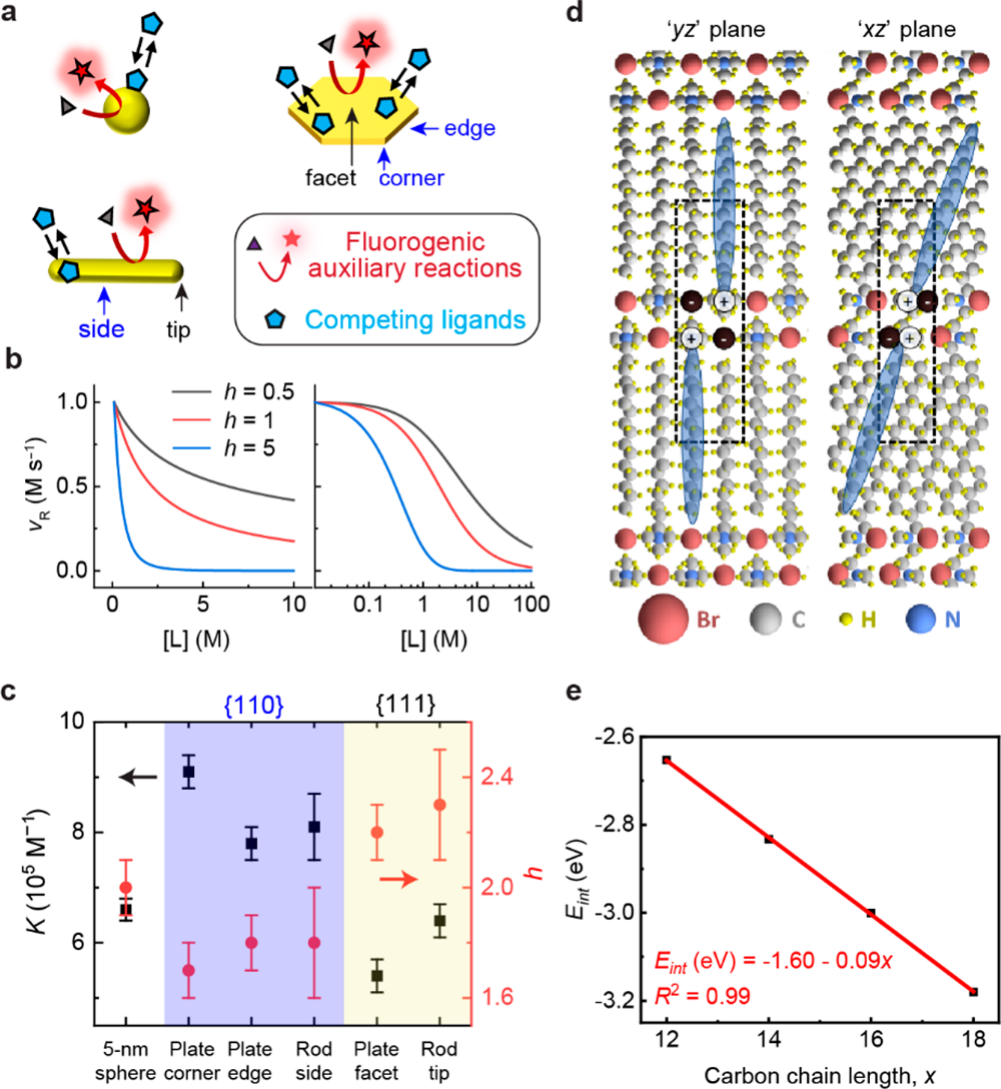
(a–c) Overview of studying adsorption affinity and cooperativity of *x*TAB on Au nanoparticles. (a) Schematic of the principle of COMPEITS. The ligand under study and the reactant of the fluorogenic auxiliary reaction compete for the same surface sites on nanoparticles (spheres, nanoplates, or nanorods), where the reaction rate of the fluorogenic reaction is monitored or imaged as a function of the competing ligand concentration. The dominant facet of structural parts of the nanoplate and nanorod are color-coded: blue denotes {110} and black denotes {111}. (b) Simulated plots of reaction rate *v*_R_ as a function of ligand concentrations [L] in a linear–linear (left) or linear–logarithmic scale (right) with different Hill coefficient *h* representing positive (*h* = 5), negative (*h* = 0.5), or no (*h* = 1) cooperativity. (c) Summary of adsorption equilibrium constants (*K*) and Hill cooperativity coefficients (*h*) of CTAB determined on pseudospherical 5 nm Au nanoparticles; at the corner, edge, and flat facet regions of Au nanoplates (see cartoons in a); and at the tips and sides of Au nanorods. The corner/edge regions of the nanoplate and the side-facets of Au nanorods are dominated by {110} facets, while the flat facets of nanoplates and the tips of nanorods are dominated by {111} facets. Error bars are standard error of the mean (sem) from many individual nanoparticles. Data from reference (18). (d–e) Self-interaction energies of *x*TAB. (d) Energy-minimized calculated crystalline structure of CTAB. Two side views from the *yz* and *xz* planes are shown. The blue shades denote the alkyl tail group. Circles denote the ammonium cation (“+”) and the bromide anion (“–”). The dashed lines denote the unit cell. Two CTAB molecules are present per unit cell. (e) Calculated interaction energy (*E*_int_) per *x*TAB molecule in its own crystalline lattice as a function of the number of carbon atoms (*x*) in the alkyl tail group. CTAB has a carbon number of 16. The red line indicates the result from the linear regression.

Recently, we reported a study of quantitative adsorption behaviors of cetyltrimethylammonium bromide (CTAB) on individual Au nanoparticles of various morphologies *in situ* under ambient solution conditions.^18^ We chose Au nanoparticles for their wide applications in catalysis,^19−22^ biolabeling,^23,24^ and sensing,^24−26^ and CTAB because it is a ligand widely used in Au nanoparticle synthesis, stabilization, and surface modification.^27−29^ Several groups have pioneered the shape-controlled synthesis of gold nanoparticles using CTAB and other ligands, including Murphy,^30^ El-Sayed,^31^ Liz-Marzan,^32^ and Mulvaney.^33^ Notably, Murphy and co-workers found that as the surfactant chain length increased, the aspect ratio of the resulting gold nanoparticles increased,^30^ suggesting that the chain length can influence the ligand’s relative adsorption among different facets. Recently, Murphy, Huang, and co-workers directly visualized and quantified CTAB distributions on gold nanorods using electron energy loss spectroscopy in an aberration-corrected scanning transmission electron microscope.^34^ They observed a higher density of CTAB at the ends of nanorods than on the sides. However, in these studies, the CTAB concentration ([CTAB]) exceeded the critical micelle concentration^35^ (0.96 mM at 25 °C) under synthesis conditions (e.g., at ∼0.10 M in Murphy’s work^30^ and El-Sayed’s work^31^) or during the sample drying process. Relatedly, based on surface-enhanced Raman spectroscopy, Hafner proposed the transition between a highly ordered bilayer, which requires a high flux of CTAB micelles to maintain the structure, to a collapsed bilayer when [CTAB] decreased from 5 to 2 mM.^36^ The adsorption behaviors of CTAB under lower concentrations, e.g., during their applications, are less explored. In our previous work, we used COMPetition-Enabled Imaging Technique with Super-resolution (COMPEITS)^18,37^ to spatially resolve adsorption behaviors between different facets on individual particles at ∼20–40 nm resolution. COMPEITS is based on competitive adsorption that suppresses the rate of a surface-catalyzed fluorogenic auxiliary reaction (Figure 1a), where the rate of the fluorogenic reaction, *v*_R_, follows eq 1 (Figure 1b):
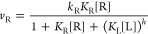
1Here, *k*_R_ is a (specific) rate constant; *K*_R_ and *K*_L_ are the adsorption equilibrium constants of the reactant (R) of the fluorogenic auxiliary reaction, which follows the Langmuir–Hinshelwood mechanism, and of the ligand (L), respectively. *h* is the Hill coefficient of cooperativity:^38−41^*h* > 1 for positive cooperativity of ligand adsorption where adsorbed ligands exhibit attractive interactions between each other;^40^*h* < 1 for negative cooperativity where the adsorbates exhibit repulsive interactions; and *h* = 1 for Langmuir adsorption, which is noncooperative (i.e., negligible interactions among adsorbates). Both this fluorogenic auxiliary reaction and its suppression can be imaged via single-molecule fluorescence localization microscopy,^18,37,42−46^ giving the superoptical resolution of mapping the competitor adsorption on the catalyst surface. COMPEITS also selectively probes the first-layer adsorption of the competing ligand, as multilayer adsorption does not lead to further suppression of the fluorogenic auxiliary reaction. Moreover, the same competition concept and the associated eq 1 can be applied in bulk measurements to quantify adsorption, albeit spatial resolution is no longer obtainable, and the results would then reflect the average properties of the particles in the bulk sample.

By examining individual pseudospherical 5 nm Au nanoparticles as well as the different regions on single Au nanoplates or nanorods that expose {110} and {111} surface facets, we found that CTAB was adsorbed more strongly on Au {110} facets than on {111} facets (i.e., adsorption equilibrium constant *K*^{110}^ > *K*^{111}^) (Figure 1c, black points).^18^ Moreover, we discovered that CTAB adsorption on Au nanoparticle surfaces exhibits positive cooperativity, with a Hill cooperativity coefficient of *h* > 1 (Figure 1c, red points). More interestingly, the positive cooperativity also differs in extent on different facets with the stronger adsorbing facet {110} showing weaker positive cooperativity (i.e., smaller *h*; Figure 1c, blue-vs-yellow shaded regions). We attributed the positive cooperativity of CTAB adsorption to the attractive hydrophobic interactions between the alkyl chains of the cetyltrimethylammonium cation (CTA^+^) in the standing-up adsorption configuration as in self-assembled monolayers. But the molecular basis for CTAB’s affinity differences between the two facets and the associated different extents of cooperativity remains unclear. Additionally, we discovered that when using CTAB as a stabilizing ligand across a range of ligand concentrations in colloidal synthesis of Au nanoparticles, the nanoparticles’ exposed {110} are more stabilized at low [CTAB], but their {111} facets are more stabilized at high [CTAB], giving rise to an adsorption crossover behavior of CTAB between different surface facets.^18^

Here, we report a combined experimental and computational study of the adsorption behaviors of alkyl-trimethylammonium bromides (*x*TAB) with variable alkyl chain length *x* on Au nanoparticles. We calculate *x*TAB adsorption on Au{111} and Au{110} surfaces with standing-up and lying-down configurations, which provides insights into the adsorption affinity and cooperativity differences of *x*TAB on these two facets. The computational results predict and explain the experimental discovery of *x*TAB’s adsorption behavior switch from stronger affinity, negative cooperativity to weaker affinity, positive cooperativity when *x*TAB’s concentration increases in solution; they also rationalize the adsorption crossover of CTAB between the two types of Au surface facets. These results showcase the power of combined experimental and computational approaches to gain molecular-scale insights into adsorbate–surface interactions.

## Results and Analysis

### *x*TAB Adsorption in Standing-up Configuration: Difference in Affinity between Au{111} and Au{110}

To gain molecular insights into our experimental discoveries of CTAB adsorption on Au nanoparticles,^18^ we used DFT calculations to examine the interaction of Au surfaces with *x*TAB ligands that have different alkyl chain lengths (i.e., C_*x*_H_2*x*+1_N(CH_3_)_3_Br; *x* = 16 for CTAB). As a control, we first studied *x*TAB molecules when they were packed in their own crystalline structure (Methods, Figure 1d) in the absence of any metal surfaces. The molecules are packed in a cuboid unit cell with a head-to-tail configuration. The optimized lattice constants of the unit cell for CTAB are *a* = 5.535 Å, *b* = 7.031 Å, and *c* = 26.548 Å, which are in reasonable agreement with the experimental values of *a* = 5.638 Å, *b* = 7.272 Å, and *c* = 26.007 Å.^47^ Similar lattice optimization calculations were performed for *x*TAB molecules with alkyl tail length *x* ranging from 12 to 18 carbon atoms. (An *x*TAB molecule with *x* carbon atoms in the alkyl chain is denoted by C_*x*_; the same notation is used throughout the following discussion.) Based on these calculations, we determined the interactions between *x*TAB molecules in the absence of any metal surface, defined as the total energy difference between an *x*TAB molecule in its crystalline bulk and an isolated *x*TAB molecule in the gas phase. The interactions mainly stem from (1) the electrostatic interaction between the ammonium–bromide ion pair (hydrophilic) and the alkyl tail group (hydrophobic) and (2) the van der Waals (vdW) interactions between the carbon chains in the alkyl groups. The former is independent of the molecule size, while the latter should scale roughly linearly with the alkyl chain length. To further delineate these two interactions, we evaluated the interaction energy per *x*TAB molecule and plotted the energy value as a function of the number of carbon atoms in the alkyl chain (Figure 1e). The slope of the interpolated line, which denotes the strength of the vdW stabilizing interactions in the absence of any metal surfaces, is −0.09 eV per carbon atom.

**Figure 2:**
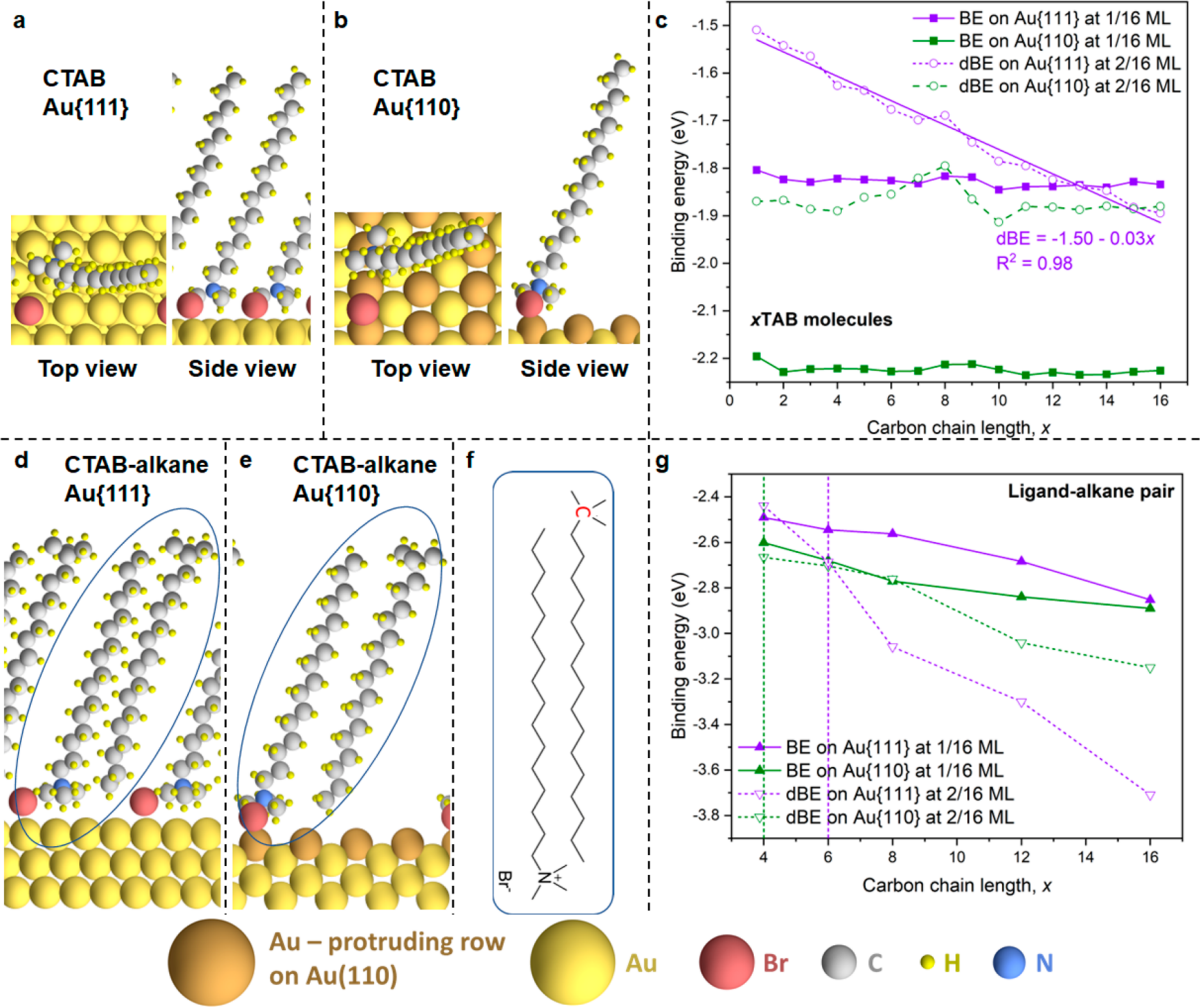
Computational studies of *x*TAB adsorption in the standing-up configuration on Au surfaces. (a–b) Lowest-energy binding structures of CTAB molecules (C_16_) in the standing-up configuration on (a) Au{111} and (b) Au{110} at 1/16 ML coverage. (c) Binding energies (BE) at 1/16 ML (solid symbols and lines) and differential binding energies (dBE) at 2/16 ML (open symbols and dashed lines) of *x*TAB molecules in the standing-up configuration on Au{111} (purple) and Au{110} (green) as a function of the alkyl chain length, *x*. The purple straight line denotes the linear regression between the dBE on Au{111} at 2/16 ML and the number of carbon atoms in the alkyl chain; the respective equation and R^2^ are shown in purple. (d–e) Optimized structure of a C_16_ ligand–alkane pair adsorbed on (d) Au{111} and (e) Au{110} at 1/16 ML coverage. The blue oval indicates a ligand–alkane pair arranged in a head-to-tail configuration. (f) The molecular structure of the C_16_ ligand–alkane pair (with no surface involved). (g) Binding energies (BE) at 1/16 ML (solid symbols and lines) and differential binding energies (dBE) at 2/16 ML (open symbols and dashed lines) of *x*TAB ligand–alkane pairs on Au{111} (purple) and Au{110} (green) as a function of the alkyl chain length. Purple and green vertical lines denote the smallest chain length required for positive adsorption cooperativity on Au{111} and Au{110}, respectively.

We then studied the adsorption of *x*TAB molecules on Au{111} and Au{110} in the fully standing-up configuration at the low-coverage limit (1/16 monolayer (ML)), where adsorbate–adsorbate interactions are expected to be insignificant. The lowest-energy binding structures on Au{111} and Au{110} for CTAB (i.e., C_16_) are shown in Figure 2a, b, and those for all *x*TAB molecules (*x* = 1–16) are summarized in Supplementary Figure 4 and Supplementary Figure 5, respectively. All molecules prefer to interact with the gold surface, with the ammonium–bromide “head” group pointing downward toward the surface, while the alkyl carbon chain points (almost) vertically upward away from the surface (Figure 2a, b). For CTAB, the binding energies of an individual molecule are −1.83 and −2.23 eV on Au{111} and Au{110}, respectively (Figure 2c; solid symbols and lines). The much stronger binding on the more open Au{110} facet is consistent with the experimentally observed stronger adsorption affinity of CTAB on Au{110} than on Au{111} (Figure 1c). We also studied the binding strength of *x*TAB molecules on both Au facets at the low-coverage limit (1/16 monolayer (ML)) as a function of the alkyl chain length (Figure 2c; solid lines and symbols); the binding energies are roughly invariant with the carbon chain length regardless of the Au facets. This indicates that, in the standing-up configuration and under low coverage, the interaction between a *x*TAB molecule and a metal surface is dominated by the headgroup, while the alkyl chain plays an almost negligible role.

To probe the interactions between *x*TAB molecules coadsorbed on the Au{111} or Au{110} surface, we evaluated the differential binding energy (dBE) of C_1_–C_16_ at 2/16 ML surface coverage (defined as the energy gained or lost when a second molecule was introduced to the surface with a molecule already adsorbed at 1/16 ML). The dBE values are also plotted as a function of the alkyl chain length in Figure 2c (open symbols and dashed lines). We note that for the C_1_ molecule (i.e., tetramethylammonium bromide), the dBE values at 2/16 ML (−1.51 and −1.87 eV on Au{111} and Au{110}, respectively) are significantly less negative than the BE values at 1/16 ML (−1.80 and −2.20 eV on Au{111} and Au{110}, respectively). This indicates that in the absence of the alkyl tail group, the interactions between the “head” groups (i.e., ammonium–bromide ion pairs), through which the *x*TAB molecules are adsorbed on the Au surfaces, are repulsive and cannot by themselves explain the positive adsorption cooperativity observed in our experiments.

We therefore examined the effect of the alkyl tail group on the interactions between *x*TAB molecules by observing the chain length dependence of the BE and dBE values (Figure 2c). On Au{111}, we observed an approximately linear dependence with a negative slope of −0.03 eV per C atom (Figure 2c, open purple symbols); also, for *x*TAB molecules larger than C_13_, the dBE at 2/16 ML becomes more negative than BE at 1/16 ML, indicating a positive cooperativity for these molecules at 2/16 ML coverage, which is consistent with experimental results on CTAB (Figure 1c, yellow shaded region). The −0.03 eV per C atom slope of this linear regression line is an indication of the interaction strength between each pair of *x*TAB molecules at 2/16 ML coverage on Au{111} due to the attractive vdW interactions between the alkyl chains, which are sufficient to offset the repulsive interactions between the head groups at *x* > 13.

On the other hand, we did not observe a strong dependence between the dBE at 2/16 ML on Au{110} and the carbon chain length; this suggests negligible vdW interactions between each pair of *x*TAB molecules adsorbed at 2/16 ML coverage on Au{110} when adsorbed in the standing-up adsorption configuration; therefore, the interactions between the adsorbed *x*TAB molecules on Au{110} remain to be dominated by the repulsive interactions between the head groups. The dBE at 2/16 ML on Au{110} is always less negative than the BE value at 1/16 ML, indicating negative cooperativity for *x*TAB molecules adsorbed at 2/16 ML coverage on Au{110}, contradicting the experimental observations on CTAB (Figure 1c, blue shaded region) and suggesting potential deficiencies in the adsorption model as in Figure 2a, b. Nevertheless, we note that the distinct interaction nature between *x*TAB molecules on Au{111} and Au{110} is due to a templating effect of the Au surface facet, which restricts the intermolecular distance between adsorbates. We also note that the intermolecular distance in the respective energy-minimized structures decreases, while the vdW interaction strength increases in the following order (Table 1): Au{110} to Au{111} to no surface. For the CTAB (C_16_) molecule, the dBE at 2/16 ML is even more negative on Au{111} than Au{110} (Figure 2c), indicating a preference for the close-packed facet at high surface coverage. Although these results have not yet fully explained the origin of the positive cooperativity of *x*TAB molecules on the more open Au{110} surface (see further results below), they shed light on the different lattice spacings, which lead to different intermolecular distances, as a possible explanation for the adsorption crossover behavior between the {110} and {111} facets when the concentration of CTAB was increased as a stabilizing ligand in colloidal Au nanoparticle synthesis.^18^

**Table 1:** Estimated Stabilizing vdW Interaction Strength Per Carbon Atom and Average Intermolecular Distance for *x*TAB Molecules in Its Own Crystalline Lattice, on Au{111}, and on Au{110} at 2/16 ML Coverage

Environment	vdW interaction per C atom (eV)^a^	Average chain–chain distance (Å)
Au{110}	∼0	10.0
Au{111}	–0.03	7.9
No surface	–0.09	6.2

a Negative sign denotes attractive interaction.

### Improved Model for *x*TAB Adsorption on Au Surfaces in the Standing-up Configuration: A Truncated Bilayer Model

Previous experimental X-ray scattering study for CTAB molecules adsorbed on a Au nanorod indicated that the molecules should be adsorbed in a head-to-tail bilayer configuration,^48^ which was supported by direct^34^ and indirect^49^ images of dried samples from transmission electron microscopy. Note that no direct imaging of the organic portion in solution is currently available. To more realistically model the adsorption of *x*TAB molecules on Au surfaces, we constructed adsorption models to account for the layered superstructure. Here, we focus on modeling a truncated bilayer structure involving *x*TAB molecules adsorbed on the Au surface paired in a head-to-tail configuration while ignoring additional molecules above this truncated bilayer, for which the interactions resemble those in a bulk liquid crystal structure. This truncation is also consistent with our COMPEITS measurement, which selectively probes the bottom adsorption layer on the surface as additional layers do not induce further suppression of the fluorogenic auxiliary reaction.^18,37^ For this truncated bilayer structure, we adopt a ligand–alkane paired structure, which involves an *x*TAB (C_*x*_) molecule vertically adsorbed on the Au surface with the ammonium–bromide pair pointing downward, and an alkane molecule paired in a head-to-tail configuration, with its ammonium–bromide pair replaced by a tertiary alkyl (C(CH_3_)_3_) group (see Figure 2d–f for detailed structures). We concluded that the elimination of the second ammonium–bromide pair in this configuration is necessary, as it leads to unphysical electrostatic interactions, which, in the actual *x*TAB adsorption superstructures, should be effectively screened by the aqueous electrolyte solution (e.g., in the COMPEITS measurement, the ionic strength is 0.042 molar, giving a Debye screening length of ∼1.5 nm, SI section 2.3).

We explored the binding properties of these ligand–alkane pairs on Au{111} and Au{110}. Here, in the subsequent discussion, we treat such a pair of molecules as a single adsorbate unit when counting the surface coverage. We evaluated its BE at 1/16 ML and dBE at 2/16 ML on both facets for C_4_, C_6_, C_8_, C_12_, and C_16_*x*TAB molecules. The results are summarized in Figure 2g. On the close-packed Au{111} facet, for C_6_ and larger *x*TAB molecules, the dBE at 2/16 ML is more negative than the BE at 1/16 ML coverage. On the more open Au{110} facet, in all cases, the 2/16 ML dBE is more negative than the low-coverage-limit BE. Therefore, we confirm that on both facets, the adsorption cooperativity for sufficiently large *x*TAB molecules should be positive when the layered adsorption superstructure is taken into account, consistent with our previous experimental observations on CTAB.^18^ This is due to the decreased average intermolecular distances in such an adsorption structure, which leads to enhanced stabilization due to vdW interactions between alkyl chains. We also note that based on Figure 2g, for C_8_ and larger *x*TAB molecules, there exist the following binding properties: (1) at 1/16 ML, the ligand–alkane pair binds stronger on Au{110} than on Au{111}; (2) at 2/16 ML, the second ligand–alkane pair binds stronger on Au{111} than on Au{110}. These results are consistent with and, more importantly, provide the molecular underpinnings of the experimentally observed adsorption crossover behavior for CTAB, whereby the Au{110} facet is preferred at low CTAB concentrations, with the Au{111} facet dominating at high CTAB concentrations.

Accordingly, our DFT study demonstrates that by properly taking into account the adsorption superstructure of *x*TAB molecules, which allows us to correctly model the vdW interactions between the alkyl chains, we can successfully explain the experimentally observed adsorption properties in the standing-up configuration regime. The results highlight the importance of the templating effect of the metal substrate; i.e., the Au surface facet defines the intermolecular distance between *x*TAB adsorbates and thereby determines the strength of vdW interactions. Our results shed light on the underlying principle behind the role of *x*TAB ligands in the facet-selective synthesis of metal nanoparticles, and hint at a potential means of controlling *x*TAB adsorption properties by tuning the substrate lattice parameters (e.g., through facet control or alloying).

### *x*TAB Adsorption on Au{111} vs Au{110} in the Lying-down Configuration: Predicting Negative Cooperativity with Stronger Affinity

**Figure 3:**
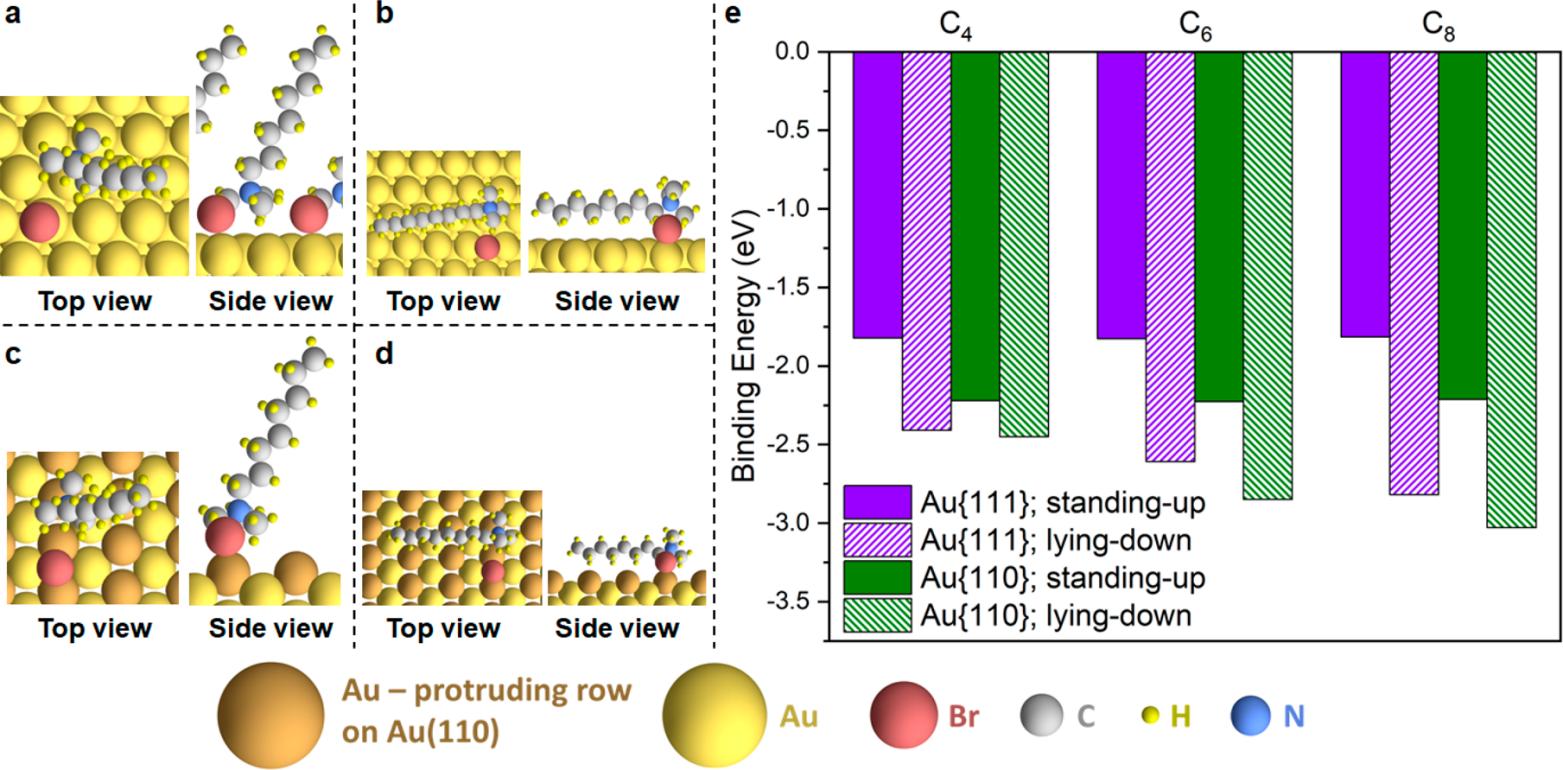
Computational studies of *x*TAB adsorption in the standing-up vs lying-down configurations on Au surfaces. (a–b) Lowest-energy binding structures of C_8_*x*TAB molecule on Au{111} in the (a) standing-up and (b) lying-down configurations at the low-coverage limit (1/16 ML for the standing-up configuration; 1/32 ML for the lying-down configuration; the same definition for the low-coverage limit applies for Au{110}). (c–d) Lowest-energy binding structures of C_8_*x*TAB molecule on Au{110} in the (c) standing-up and (d) lying-down configurations at the low-coverage limit. (e) Comparison of calculated binding energies of C_4_, C_6_, and C_8_*x*TAB molecules on Au{111} and Au{110} in the standing-up and lying-down configurations at the low-coverage limit.

We also studied the adsorption of *x*TAB molecules on Au{111} and Au{110} in the fully lying-down configuration (Figure 3a–d), which is expected to happen under very low ligand concentrations, where very few ligands are adsorbed on the surface. Focusing on C_4_, C_6_, and C_8_ as representatives, their lowest-energy binding structures on Au{111} and Au{110} are summarized in Supplementary Figure 6 and Supplementary Figure 7, respectively, with the associated binding energies listed in Table 2. To ensure sufficient lateral separation between *x*TAB molecules in the lying-down configuration at the low-coverage limit, we adopted a larger unit cell, which corresponds to 1/32 ML coverage for a single adsorbate in the unit cell; this is a lower coverage than the standing-up adsorption case (1/16 ML). The average distances between the ammonium–bromide head groups at the low-coverage limit therefore increase from 11.6 and 14.0 Å to 17.7 and 22.6 Å on Au{111} and Au{110}, respectively, going from standing-up to lying-down adsorption configurations. We note that in the lying-down configuration, the alkyl chain of the *x*TAB molecules is parallel to the surface; in our fixed-size unit cell, the spacing between the head (ammonium–bromide ion pair) and tail (alkyl chain) groups decreases as the alkyl chain length increases. Therefore, a direct comparison of results for C_4_, C_6_, and C_8_ in this section should be made with caution. The focus of our work is mainly to determine qualitatively the nature of the interactions (attractive or repulsive) between the *x*TAB molecules adsorbed in the lying-down configuration.

**Table 2:** Calculated Binding Energies (BE; 1/32 ML), Differential Binding Energies (dBE; 2/32 ML), and Interaction Energies (*E*_int_; See Footnotes) of C_4_, C_6_, and C_8_*x*TAB Molecules in the Lying-down Configuration on Au{111} and Au{110}

*x*TAB	BE (eV; 1/32 ML)	dBE (eV; 2/32 ML)	*E*_int, gas_ (eV)^a^	*E*_int, surf_ (eV)^b^	*E*_int, tot_ (eV)^c^
	**Au{111}**				
C_4_	–2.41	–2.05	–0.20	0.56	0.36
C_6_	–2.61	–2.32	–0.16	0.45	0.29
C_8_	–2.82	–2.63	–0.42	0.61	0.19
	**Au{110}**				
C_4_	–2.45	–2.38	0.03	0.04	0.07
C_6_	–2.85	–2.62	0.03	0.20	0.23
C_8_	–3.03	–2.85	0.02	0.16	0.18

a *E*_int, gas_ = *E*_2*x*TAB_ (*fixed*) – 2*E*_gas_, where *E*_2*x*TAB_ (*fixed*) is the total energy of two *x*TAB molecules fixed at their corresponding positions when coadsorbed in the lying-down configuration at 2/32 ML, calculated in the absence of the Au surface, and *E*_gas_ is the total energy of an isolated *x*TAB molecule in the gas phase. *E*_int, gas_ denotes the contribution from *ligand–ligand* interaction in the absence of the Au surface to the total interaction energy (*E*_int, tot_) between two *x*TAB molecules coadsorbed at 2/32 ML in the lying-down configuration.

b *E*_int, surf_ = *E*_int, tot_ – *E*_int, gas_. *E*_int, surf_ denotes the contribution from *ligand–surface* interaction to the total interaction energy between two *x*TAB molecules coadsorbed at 2/32 ML in the lying-down configuration.

c *E*_int, tot_ = dBE (2/32 ML) – BE (1/32 ML). *E*_int, tot_ denotes the total interaction energy between two *x*TAB molecules coadsorbed at 2/32 ML in the lying-down configuration.

According to the results in Table 2, at the low-coverage limit, all three *x*TAB molecules bind more strongly on Au{110} than on Au{111}. This is the same trend as observed for *x*TAB molecules adsorbed in the standing-up configuration and can be attributed to the stronger interaction between the ammonium–bromide “head” group and the more open {110} facet (i.e., more undercoordinated surface atoms) than the closely packed {111} facet. Importantly, at the low-coverage limit (1/16 ML for standing-up configuration; 1/32 ML for lying-down configuration) and compared on a per-molecule basis, we always predict stronger binding for an *x*TAB molecule in the lying-down configuration than the standing-up configuration on both Au{111} and Au{110} (Figure 3e). This is consistent with our experimental discovery below that the adsorption affinity is always stronger for the lying-down configuration in the low-concentration regime on both facets. Additionally, in all cases, the dBE at 2/32 ML is less negative than the BE at 1/32 ML coverage, which indicates that the interaction between two *x*TAB molecules in the lying-down configuration is repulsive in nature and should lead to negative adsorption cooperativity. To the best of our knowledge, such negative adsorption cooperativity for *x*TAB molecules has not been observed experimentally. More excitingly, our DFT study suggests that by adjusting the surface *x*TAB coverage, one could potentially alter the sign of its adsorption cooperativity by changing *x*TAB’s adsorption configuration (i.e., standing-up vs lying-down) (see experimental discovery below).

To further elucidate the origin behind this repulsive interaction, we delineated the total interaction energy between *x*TAB molecules (C_4_, C_6_, and C_8_) adsorbed in the lying-down configuration (*E*_int, tot_; Table 2) into two contributions: (i) *E*_int, gas_, the contribution from purely ligand–ligand interactions in the absence of the Au surface; and (ii) *E*_int, surf,_ the contribution from ligand–surface interactions (see footnote of Table 2 for detailed definitions). Interestingly, we observed distinct behaviors between Au{111} and Au{110}. On Au{110}, even at 2/32 ML coverage, *x*TAB molecules adsorbed in the lying-down configuration are separated by an average distance of at least 10 Å. There exists no vdW interaction between *x*TAB molecules at this separation, as evidenced by the very small, positive *E*_int, gas_ values on Au{110} (0.02–0.03 eV; Table 2). In this case, the interaction between two *x*TAB molecules adsorbed in the lying-down configuration on Au{110} is dominated by the positive (repulsive) interaction mediated by the Au surface (*E*_int, surf_; Table 2). On Au{111}, due to the difference in lattice spacing, *x*TAB molecules coadsorbed in the lying-down configuration at 2/32 ML are packed with much smaller spacing (∼7 Å), where vdW interactions can be significant in the absence of the Au surface. This is evidenced by the negative, stabilizing *E*_int, gas_ values (−0.20 eV, −0.16 eV, and −0.42 eV for C_4_, C_6_, and C_8_, respectively; Table 2). However, the negative *E*_int, gas_ values are offset by more positive *E*_int, surf_ values (ligand–ligand interaction, mediated by the Au surface), leading to overall positive (repulsive) total interaction energies between *x*TAB molecules adsorbed in the lying-down configuration on Au{111} (Table 2). These results suggest that the interactions between *x*TAB molecules adsorbed on Au surfaces in the lying-down configuration are largely dominated by contribution from ligand–surface interactions, which are repulsive in nature. Additionally, any vdW interactions between lying-down *x*TAB molecules, even at close lateral distances, are effectively screened by the presence of the Au surface.

### Concentration-Dependent Switching of CTAB Adsorption Cooperativity

The above computational results provided insights into the standing-up adsorption configuration of *x*TAB on Au surfaces, especially the bilayer adsorption (Figure 2), which can give rise to positive adsorption cooperativity and differential affinity between the Au{111} and Au{110} facets, rationalizing our previous experimental observations on CTAB.^18^ Moreover, our computational results pointed to the possibility of lying-down adsorption configuration for *x*TAB (Figure 3 and Supplementary Figure 6 and 7), which is characterized by repulsive interactions among the adsorbed *x*TAB molecules. This repulsive interaction should lead to a negative adsorption cooperativity and should occur at lower coverages due to the much larger adsorption footprint of the lying-down configuration and lower ligand concentration in the solution because of its predicted larger adsorption energy than that of the standing-up configuration (Figure 3e). Whether this negative adsorption cooperativity indeed exists for *x*TAB is not known experimentally nor is the switching of adsorption cooperativity as a function of surface coverage. Experimental verifications are needed here, especially considering that our computational studies were based on surrogate models for the layered adsorption superstructure of *x*TAB molecules and omitted solvent and entropy effects due to constraints of the system size and the associated computational cost.

Therefore, to directly probe this potential lying-down adsorption configuration, we expanded the concentration range of CTAB adsorption in our COMPEITS titration experiments (previously lowest at 0.5 μM).^18^ We chose to perform competition titration at the bulk level because of its easier access to more experimental solution conditions. We also focused on the 5 nm Au nanoparticles as the representative because they are relatively homogeneous in size and shape, whereas Au nanoplate and nanorod samples are mixtures of particles with different morphologies (which was not a problem for our earlier COMPEITS imaging experiments that resolved individual particles). The fluorogenic auxiliary reaction is the reduction of resazurin to the highly fluorescent resorufin (Supplementary Figure 1a) that we used previously.^18^

**Figure 4:**
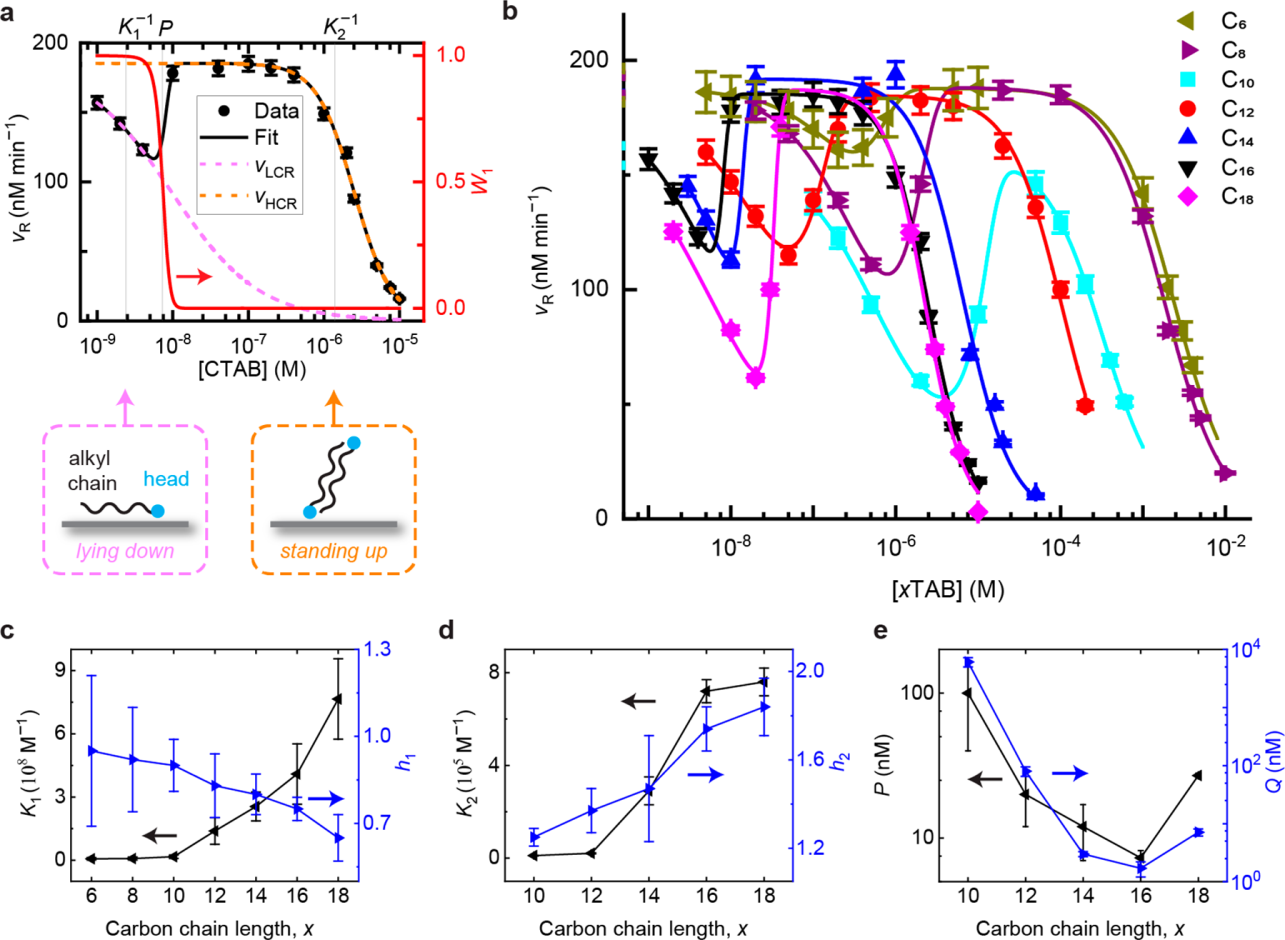
Competition titrations reveal adsorption switching of *x*TAB ligands between high-affinity negative cooperativity and low-affinity positive cooperativity on Au nanoparticles with increasing [*x*TAB]. (a) Black points: competition titration of the initial rates of the fluorogenic auxiliary reaction with increasing [CTAB]. Magenta and orange dashed lines: fits of low and high concentration regimes with eq 1, respectively. Black line: fit with eq 2. Red line: the weighting factor *W*_1_ vs [CTAB] from fitting with eq 2. (b) Same as (a) but for the series of *x*TAB ligands (C_*x*_H_2*x*+1_N(CH_3_)_3_Br). Solid lines: fits with eq 2. (c–e) The fitted *K*_1_ and *h*_1_ (c), *K*_2_ and *h*_2_ (d), and *P* and *Q* (e) as a function of *x* from b. Error bars are s.d. from 3 independent measurements (a–b) and 95% confidence bounds in fitting (c–e).

Strikingly, we found that the titration result (the initial rate *v*_R_ of the fluorogenic auxiliary reaction vs CTAB concentration [CTAB]) shows a multiphasic dependence over many orders of magnitude of [CTAB] (black circles in Figure 4a). Specifically, *v*_R_ first decreases with increasing [CTAB] in the range of 10^–9^ to 10^–8^ M, as expected for adsorption competition between CTAB and the fluorogenic auxiliary reaction, which follows the Langmuir–Hinshelwood mechanism on Au nanoparticle surfaces.^18,50^ However, *v*_R_ later increases, signaling a switch of the adsorption behavior of the competing CTAB; it then decreases again at [CTAB] values higher than 10^–7^ M until it eventually approaches zero.

To account for the behavior of *v*_R_ across the entire concentration range of CTAB, we used a modified form of eq 1 to empirically combine two different ligand adsorption behaviors as a function of its concentration (eq 2):
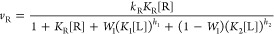
2Here, *K*_1_ (*K*_2_) and *h*_1_ (*h*_2_) are the respective adsorption equilibrium constant and Hill coefficient at the low (high) concentration regime; *W*_1_ and 1 – *W*_1_ are the weighting factors, where *W*_1_ follows an asymptotic function (eq 3):
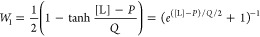
3When [L] → 0, *W*_1_ → 1; when [L] → ∞, *W*_1_ → 0. At [L] = *P*, a critical concentration that divides the low and high concentration regime behaviors, *W*_1_ transitions from 1 to 0 across a concentration width defined by *Q* (Figure 4a, red line).

Eq 2 satisfactorily accounts for the competition titration across the entire [CTAB] range (Figure 4a, black line), giving *K*_1_ = (4 ± 1) × 10^8^ M^–1^, *h*_1_ = 0.75 ± 0.04, *K*_2_ = (7.2 ± 0.5) × 10^5^ M^–1^, and *h*_2_ = 1.7 ± 0.1. More importantly, the high affinity, low concentration regime adsorption behavior (i.e., *K*_1_) has negative cooperativity (i.e., *h*_1_ < 1), confirming the computational predictions on the lying-down adsorption configuration (Table 2, Supplementary Figure 6 and Supplementary Figure 7). At concentrations higher than *P* = 7.3 ± 0.9 nM, CTAB adsorption switches to low affinity (i.e., *K*_2_) with positive cooperativity (i.e., *h*_2_ > 1), which was previously observed^18^ and shown by the above computational results to associate with the standing-up adsorption configuration (Figure 2). In this regime, the adsorption affinity (*K*_2_) and cooperativity (*h*_2_) are comparable to the reported value in our previous work.^18^ To our knowledge, such switching behavior of adsorption affinity and cooperativity is first-of-its-kind, and for CTAB, this switching occurs over a narrow concentration width (*Q* = 1.7 ± 0.5 nM), suggesting a cooperative change in the adsorption configuration from lying-down to standing-up among the adsorbed CTAB molecules on the Au nanoparticle surface.

### Alkyl-Chain Length Dependence of Adsorption Cooperativity Switching

The above computational results also predicted that the adsorption affinity and cooperativity of *x*TAB molecules should show systematic dependences on the alkyl carbon chain length, including both the standing-up and lying-down adsorption configurations. To experimentally probe such dependences, we further measured the adsorption behaviors on 5 nm Au nanoparticles of *x*TAB ligands with different alkyl chain lengths, i.e., C_*x*_H_2*x*+1_N(CH_3_)_3_Br with *x* ranging from 6 to 18, that are commercially available (Figure 4b). For *x* = 1, C_1_, i.e., tetramethylammonium bromide, is not adsorbed competitively with the reactant of the fluorogenic auxiliary reaction on Au nanoparticles (Supplementary Figure 3) and thus is not further investigated. Starting from C_6_ for *x* = 6, their competition titration curves all show the general multiphasic behaviors vs *x*TAB concentration in the aqueous solution ([*x*TAB]) similar to CTAB (i.e., C_16_), and all of them can be described satisfactorily by eq 2, reflecting their switching of adsorption behavior with increasing concentration. The fitted parameters are summarized in Supplementary Table 1.

For the low-concentration regime, high affinity adsorption, the affinity (*K*_1_) increases with the alkyl chain length *x* (Figure 4c, black points), attributable to larger vdW interactions between the longer chains and the Au surface in a lying-down adsorption configuration, as shown by the larger negative BE of the computational results on *x*TAB (Figure 3e, Table 2; Supplementary Figure 6 and Supplementary Figure 7). The Hill coefficients (*h*_1_) are all <1 across all *x*TAB ligands (Figure 4c, blue points), i.e., negative adsorption cooperativity, consistent with the computational results on net repulsive interactions between adsorbed *x*TAB molecules with lying-down configuration (Table 2). Interestingly, with increasing alkyl chain length *x*, *h*_1_ is increasingly smaller than 1 (Figure 4c, blue), indicating larger repulsive interactions between longer-chain *x*TABs. This trend also suggests that the repulsive interactions are strongly dependent on the size of the *x*TAB molecule.

For the high concentration regime, low affinity adsorption, the affinity (*K*_2_) also increases with *x* (Figure 4d, black points), suggesting that the measured adsorption strength has contributions from the alkyl chain, and agreeing with the computed standing-up bilayer adsorption configuration, which has larger negative BE for longer chains due to more attractive interactions between the alkyl chains (Figure 2d). The Hill coefficients (*h*_2_) are all >1 and further increase with increasing *x* (Figure 4d, blue points), consistent with stronger attractive interactions between longer alkyl chains. It should be noted that the measured *K*_2_ for C_6_ or C_8_ appear to be smaller than the adsorption affinity of the counteranion Br^–^ (*K*_Br^–^_ = (1.4 ± 0.2) × 10^3^ M^–1^, Supplementary Figure 2), making these two measurements unreliable; so the high concentration regime behaviors of C_6_ and C_8_ adsorption were excluded in our analysis.

The critical concentration *P*, where the adsorption behavior switches, decreases in general for ligands with longer chains, manifested in the shift of the local minima to lower concentrations in the titration curves (Figure 4b) and summarized in Figure 4e. This can be rationalized by that *P* is greater than *K*_1_^–1^, the corresponding dissociation constant for the high affinity adsorption, which is in concentration units (Figure 4a, top axis). As *K*_1_^–1^ decreases when *x* increases, *P* decreases accordingly. In other words, switching occurs with increasing [*x*TAB], which increases overall density of *x*TAB and decreases the interadsorbate distance. Eventually, it is the balance of adsorption energetics difference between lying-down and standing-up configurations, including their interadsorbate interactions, that drive the switching. Moreover, since the adsorption switching is defined between (i.e., bracketed by) the low-concentration, high-affinity adsorption and the high-concentration, low-affinity adsorption (i.e., it occurs between *K*_1_^–1^ and *K*_2_^–1^), the transition width *Q* must be smaller than (*K*_2_^–1^ – *K*_1_^–1^), which has a general decreasing trend with increasing *x*. Indeed, *Q* is generally smaller for larger *x* (Figure 4e, blue points), and the ligands with longer chains show sharper transitions in the competition titration curves (Figure 4b). For C_6_ and C_8_, the high-concentration, low-affinity binding is dominated by the bromide, not the *x*TA^+^. Therefore, both the switching concentration (represented by *P*) and the transition width (represented by *Q*), can still be fitted from eq 2 as shown in Figure 4b, but these fitted values do not physically reflect the lying-down switching to standing-up of the *x*TA^+^ chain and thus are not reliable. Therefore, C_6_ and C_8_ data points of the switching or the high-concentration, low-affinity binding are excluded in Figure 4d and 4e. We note that for C_18_, its critical concentration *P* for adsorption switching and its transition width *Q* are both higher than those of the general trends (Figure 4e). This outlying behavior of C_18_ could perhaps originate from that with a long enough alkyl chain, the switching of adsorption configuration would involve larger range molecular motions and larger entropy changes, or perhaps that for such a long chain, the chain tail could “turn around” and interact with the earlier part of the same tail-chain; the exact nature remains to be investigated.

## Concluding Remarks

Through a combination of experimental and computational studies, we have gained a molecular-level understanding of the adsorption behaviors of alkyl-trimethylammonium bromides (*x*TABs), a key class of ligands for colloidal nanoparticle stabilization and shape control, on Au nanoparticle surfaces. We found that in both the standing-up and lying-down adsorption configurations, *x*TABs were adsorbed stronger on Au{110} than on Au{111}, which resulted mainly from the stronger ammonium–bromide headgroup adsorption in both the standing-up configuration and the lying-down configuration. In general, compared with the standing-up configuration, the lying-down configuration has much stronger adsorption and larger intermolecular distance and shows repulsive intermolecular interactions instead of attractive interactions. The standing-up configuration enables a densely packed multilayer structure for *x*TAB molecules adsorbed on Au surfaces; the close distances between the alkyl chains result in large attractive vdW forces which dominate the adsorbate–adsorbate interactions. For *x*TAB molecules adsorbed in the lying-down configuration, vdW interactions can only contribute to the binding strength of an individual ligand; ligand–ligand vdW interactions, even if they exist, are effectively screened by the presence of the Au surface, and repulsive lateral interactions dominate. These together underlie the behavior switch from a higher affinity, negative cooperativity adsorption, to a lower affinity, positive cooperativity adsorption when the concentration of *x*TABs in the solution phase increases. Additionally, in the standing-up configuration, bilayer adsorption may occur on both facets. Due to the templating effect of the metal substrate, which defines interaction strength between *x*TAB molecules, the different lattice spacing between Au{110} and Au{111} gives rise to distinct differential binding energies and consequently adsorption crossover between the two facets when the ligand concentration increases.

The examples and insights described here showcase the power of combining experimental and computational approaches to gain molecular-scale insights into adsorbate–surface interactions. They also point to further opportunities in examining the adsorption behaviors of other ligands widely used in nanoparticle synthesis and stabilization, for example, poly-*N*-vinylpyrrolidone (PVP), thiols, and other micelle systems. However, challenges still remain, such as the size of PVP, which exceeds the typical reach of current DFT calculations, the effects of solvent and electrolyte, and the contributions of entropy besides electronic energies. Nonetheless, valuable insights can be gained through a judicial selection of model systems, as demonstrated in this study.

## Methods

### UV–Vis Measurements to Monitor Bulk Reaction Kinetics

UV–vis absorption measurements were used to monitor the consumption of the reactant resazurin of the auxiliary fluorogenic reaction (i.e., reduction of resazurin by NH_2_OH at pH ∼ 7.3; details in Supporting Information Section 1) to determine the reaction rate. The UV–vis absorption spectra were obtained with a Beckman Coulter DU 800 spectrometer at room temperature (20 °C). A spectrum in the 550–650 nm window was collected every 30 s during the titration experiment at a scan rate of 600 nm per minute; 10 spectra in 5 min are collected to evaluate the initial rates of the fluorogenic auxiliary reaction. The collection of spectra rather than simply monitoring a specific wavelength over time was to ensure that the change in the spectrum was indeed caused by the reduction of resazurin catalyzed by Au nanoparticles and not by background drifting or other experimental imperfections. The concentrations of resazurin [R] were extracted from the absorbance at 602 nm for the calculation of reaction rates (denoted in *v*_R_), using the extinction coefficient ε_602_ = 56000 M^–1^ cm^–1^. A titration at each set of conditions was performed 3 times, in which each set of data was treated independently to obtain the initial rate; the average and standard deviation of such 3 initial rates were plotted (e.g., in Figure 4 and Supplementary Figures 1c–e) and used for analysis to extract the adsorption parameters of the competitor. No unexpected or unusually high safety hazards were encountered.

The fluorogenic auxiliary reaction and *x*TAB are unlikely to affect each other beyond competitive adsorption on the gold surface for the following reasons. First, [*x*TAB] is generally low and always below the critical concentration of micelle formation, i.e., *x*TAB is completely dissolved in water as isolated ions, so it is unlikely that the reactant may be sequestered by the free *x*TAB molecules. Second, the reactant, resazurin, and the product, resorufin, of the fluorogenic auxiliary reaction are highly hydrophilic anions. If resazurin and resorufin were sequestered by *x*TAB, they would have a tendency to form aggregates and their absorption/fluorescence spectra should change, but we do not see such changes experimentally. Lastly, there could be other hidden equilibria that we did not include in our kinetic model, but our model is intended to be a minimal one that sufficiently describes all the data.

### Bulk Competition Titration

Bulk competition experiments were performed based on the titration of catalytic activities of Au nanoparticles in the absence (and then presence) of ligands. Specifically, pseudospherical colloidal Au nanoparticles, 5 nm in diameter nominally, were used to catalyze the reduction of resazurin (R) to resorufin by NH_2_OH (Supplementary Figure 1a), which was provided in excess, and monitored by UV–vis absorption spectrometry (Supplementary Figure 1b). For all competitive adsorption titrations shown in Figure 4b, the reactant of the fluorogenic auxiliary reaction, namely, resazurin, is held at a constant concentration [R] = 10.0 μM. From eq 1 and 2, the [R] term appears in both numerator and denominator, but the *K*_L_’s and *h*’s only depend on the titration behavior vs [L]. In other words, the titration can be performed at any [R], which, in principle, will give the same result in determining *K*_L_ and *h*. Practically, [R] affects the magnitude of the measured fluorogenic rate, but does not change the shape of the titration curve vs [L].

### Computational Details

Periodic DFT calculations were performed using the Vienna ab initio software package (VASP).^51,52^ The exchange-correlation functionals were described by the generalized gradient approximation (GGA-PBE).^53^ Long-range dispersive interactions, which are poorly described by PBE alone, were accounted for using Grimme’s D3 method.^54−56^ The electron–ion interactions were described using the projector augmented-wave (PAW) potentials,^57,58^ and the Kohn–Sham electron wave functions were expanded using plane-wave basis sets with a kinetic-energy cutoff of 400 eV. For calculations with *x*TAB molecules in their own crystalline lattice, periodic structures similar to those reported by Almora-Barrios and co-workers were used^59^ (Figure 2d). The first Brillouin zone of each unit cell was sampled with a (6 × 6 × 1) Monkhorst–Pack k-point mesh.^60^ For surface adsorption calculations, each Au surface (either {111} or {110}) was modeled by a three-layer slab infinitely repeated in a supercell geometry with the bottom two atomic layers of the metal slab fixed at their truncated bulk lattice positions. For *x*TAB molecules adsorbed in the “standing-up” configuration, a (4 × 4) surface supercell was adopted; for “lying-down” adsorption configurations, an (8 × 4) supercell was used. Adsorption was allowed on only one side of the metal slab, and the electrostatic potential was adjusted accordingly.^61,62^ Any pair of successive slabs in the surface norm direction was separated by a vacuum layer of varying thickness, which allows for at least 10 Å of separation between the top of the adsorbate molecule and the bottom of the next mirror-image slab. The first Brillouin zone of each unit cell was sampled with a (4 × 4 × 1) Monkhorst–Pack k-point mesh for the (4 × 4) surface supercell and a (2 × 4 × 1) mesh for the (8 × 4) supercell. The calculated lattice constant for Au is 4.100 Å, which is in good agreement with the experimental value of 4.078 Å.^63^

For a single *x*TAB molecule, its binding energy (BE) is defined as
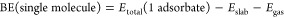
where *E*_total_(1 adsorbate) is the total energy of an *x*TAB molecule adsorbed on a Au slab; *E*_slab_ is the total energy of a clean Au slab; *E*_gas_ is the total energy of an isolated *x*TAB molecule in the gas phase. The differential BE (dBE) of an *x*TAB molecule at a higher coverage is defined as
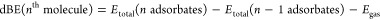
where *E*_total_(*n* adsorbates) and *E*_total_(*n* – 1 adsorbates) denote the total energies of an Au slab with *n* and *n* – 1 *x*TAB molecules adsorbed on it, respectively.

For *x*TAB ligand–alkane pairs, their BE at the low-coverage limit and dBE at higher coverages are defined as
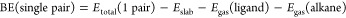

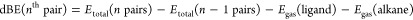
where *E*_total_(1 pair), *E*_total_(*n* pairs), and *E*_total_(*n* – 1 pairs) denote the total energies of an Au slab with 1, *n*, and *n* – 1 ligand–alkane pairs adsorbed on it, respectively. *E*_gas_(ligand) and *E*_gas_(alkane) denote the total energies of an *x*TAB ligand and an alkane molecule of equivalent carbon chain length, each isolated in the gas phase, respectively.

## Supporting Information Available

Materials and methods; titration results of potassium, phosphate, bromide, and C_1_; Debye length calculation; data table; lowest-energy binding structures of *x*TAB molecules in the standing-up or lying-down configuration on Au{111} or {110} from DFT calculations (PDF)
