# Supplementary material for: Molecular-scale Insights into Cooperativity Switching of xTAB Adsorption on Gold Nanoparticles
Source: ACS Cent Sci. 2024 Jan 4;10(1):65–76. doi: 10.1021/acscentsci.3c01075 (PMC10823513; doi:10.1021/acscentsci.3c01075)
Supplement: Supplementary file 1 — oc3c01075_si_001.pdf [file oc3c01075_si_001.pdf]

Supplementary Information

Molecular-scale Insights into Cooperativity Switching of *x*TAB Adsorption on Gold Nanoparticles

Lang Xu,[1†] Rong Ye,[2,3†] Manos Mavrikakis,[1*] Peng Chen[2*]

[†]Equal contributions

[*]Corresponding authors: M.M., emavrikakis@wisc.edu; P.C., pc252@cornell.edu

Table of Contents

1. Materials and Methods 2
2. Additional results 3
   2.1. K^+ and phosphate anions have negligible adsorption affinity to Au nanoparticle surface, while Br^- anion adsorbs non-cooperatively with $K_{Br^-} = 1.2$ mM^{-1} 3
   2.2. Competition titration shows that C_1 interacts with Au nanoparticle surface differently from other *x*TAB ($x \geq 6$) 4
   2.3. Debye length calculation 4
3. Additional figures and tables 4
4. References 7

1. Materials and Methods

Chemicals: gold colloid nanoparticles [5 nm, Ted Pella, 15702, web: https://www.tedpella.com/gold_html/goldsols.aspx (accessed Oct 6, 2023), with the Materials Safety Data Sheet therein; the solution contains trace amounts of citrate, tannic acid, and potassium carbonate, which are all weak ligands that can be washed away readily], tetramethylammonium bromide (C1, ≥98.0%, Sigma-Aldrich 426296), hexyltrimethylammonium bromide (C6, ≥98.0%, Sigma-Aldrich 53272), trimethyloctylammonium bromide (C8, ≥98.0%, Sigma-Aldrich 75091), decyltrimethylammonium bromide (C10, ≥98.0%, Sigma-Aldrich 30725), dodecyltrimethylammonium bromide (C12, ~99%, Sigma-Aldrich D5047), myristyltrimethylammonium bromide (C14, ≥99%, Sigma-Aldrich T4762), hexadecyltrimethylammonium bromide (CTAB or C16, 99+%, Thermo Scientific, AC227161000), trimethyloctadecylammonium bromide (C18, ~99%, Sigma-Aldrich 359246), and hydroxylamine hydrochloride ($NH_2OH{\cdot}HCl$, 99%, 159417) were used as received without further treatment unless otherwise noted. High purity resazurin sodium salt (~99%, Molecular Probes, Thermo Fisher Scientific, R12204) was further purified via thin layer chromatography before use. All H_2O used was purified via an Elga water purification system to reach the resistivity of 18.2 MΩ/cm.

Bulk competition titration. Bulk experiments were performed based on the titration of catalytic activities of Au nanoparticles in the absence (and then presence) of ligands. Specifically, pseudo-spherical colloidal Au nanoparticles, 5 nm in diameter nominally, were used to catalyze the reduction of resazurin (R) to resorufin by NH_2OH (Supplementary Fig. 1a), which was provided in excess. The reduction of resazurin was monitored by UV-Vis absorption spectrometry (Supplementary Fig. 1b).

Before the titration of ligands, each batch of Au nanoparticles was titrated with resazurin (R) to extract the corresponding K_R values for Eq. 1-2. Typically, 100 μL of the 5-nm Au suspension (0.010 ~ 0.10 nM based on the number of particles) was added to a premixed 7 mM phosphate buffer solution (pH = 7.4) containing different amounts of R (1.0 ~ 10 μM) and an excess amount of NH_2OH (1.0 mM). The mixture was shaken and immediately poured into a cuvette for UV-Vis measurements. The reaction mixture turned gradually from blue to red, and the absorption peak at 602 nm (from R) decreased while the absorption peak at 572 nm (from the product resorufin) increased (Supplementary Fig. 1b). The concentration of R decreases linearly (typically a linear fitting gives $R^2 > 0.99$) with respect to time in the first 10~20 min, and linear fit to give the slope that was taken as the initial reaction rate v.

For the COMPEITS titration experiments, the reaction conditions were kept the same except that [R] was fixed at 10.0 μM while the competing ligand (e.g., one of the xTAB) with increasing concentrations was added into the reaction mixture. The ligand concentrations range from nM to mM depending on the affinity of the ligand. Generally, stock solutions of xTAB in mM concentrations were prepared by dissolving a solid ligand sample (a few mg) in water (a few

mL). The most concentrated stock solutions were diluted to prepare solutions at 100-fold concentration intervals. The ligand concentration in the reaction titration experiment was achieved from adding a calculated volume (typically in a few or tens of µL) of the most suitable stock solution.

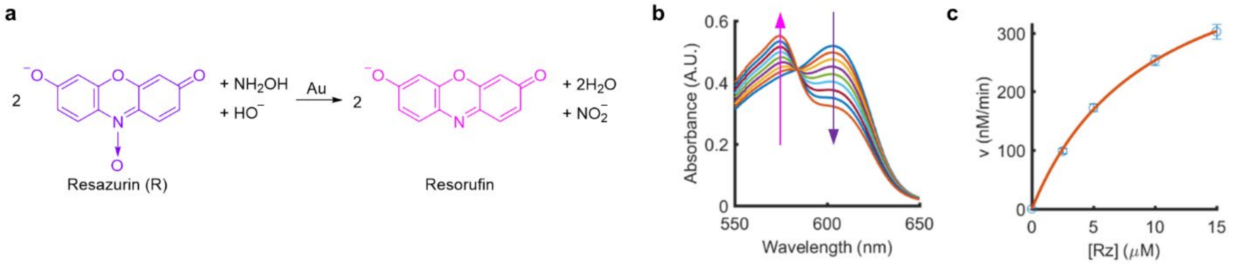

Supplementary Fig. 1. Reaction scheme and representative titration measurements for the fluorogenic reaction of resazurin reduction by NH_2OH to resorofin. a, Scheme of the fluorogenic auxiliary reaction catalyzed by gold nanoparticles. **b**, In situ absorbance measurements of the reduction of R by NH_2OH catalyzed by Au nanoparticles in an aqueous solution. Conditions: $[R]_0$ = 10.0 µM; $[NH_2OH]_0$ = 1.0 mM; [Au nanoparticle] ≈ 8.3 nM (based on particles instead of atoms), in a phosphate buffer (7 mM, pH 7.4). The blue and magenta arrows indicate the decrease of R absorption band at 602 nm and the increase of resorufin absorption band at 572 nm, respectively. The consumption of R is used to evaluate the rate of the fluorogenic auxiliary reaction. **c**, The initial reaction rates vs. the R concentration. $[NH_2OH]_0$ = 1.0 mM; [Au nanoparticle] = 8.3 nM, in 7 mM pH 7.4 sodium phosphate buffer. Red line is the fit with Langmuir kinetics $v_R = \frac{k_R K_R [R]}{1+K_R[R]}$, with k_R = 0.27 ± 0.07 µM min^{-1}, K_R = 1.7 ± 1.0 µM^{-1}.Error bars are s.d.

2. Additional results

2.1. K^+ and phosphate anions have negligible adsorption affinity to Au nanoparticle surface, while Br^- anion adsorbs non-cooperatively with K_{Br-} = 1.2 mM^{-1}

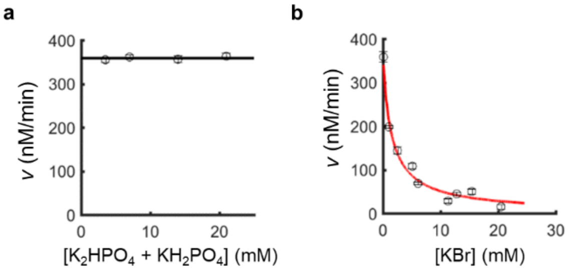

Supplementary Fig. 2. Competition titration curves and fittings of the phosphate buffer (**a**) and KBr (**b**). All conditions are the same as in Supplementary Fig. 1c other than the studied species. **a**, eye guide showing that neither the phosphate anions nor the potassium cation concentration affects the fluorogenic reaction rate, indicating they have negligible adsorption onto Au nanoparticle surface. **b**, Red line: fitting with Eq. 1., with K_{Br-} = 1.2 ± 0.3 mM^{-1} and h = 0.98 ± 0.04 (i.e., $h \approx 1$; no cooperativity). As the potassium cation is not affecting the reaction rate as shown in **a**, this curve is an effective titration of the bromide anion. Error bars are s.d.

2.2. Competition titration shows that C_1 interacts with Au nanoparticle surface differently from other *x*TAB ($x \geq 6$)

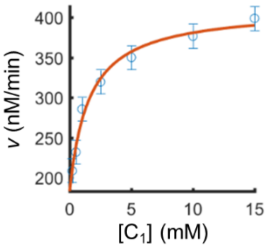

Supplementary Fig. 3. Titration curve and fitting of tetramethylammonium bromide (C_1). All conditions are the same as in Supplementary Fig. 1c other than the addition of C_1. The fluorogenic reaction rate increases with [C_1], indicating somehow C_1 does not adsorb onto Au nanoparticles competitively with the reactant resazurin of the fluorogenic auxiliary reaction and suppress the reaction rate, but appear to promote the reaction rate. Therefore, the competition kinetics as described by Eq (1) cannot be applied here. An empirical saturation fitting is used to evaluate the apparent adsorption affinity of C_1 in such promoting effect, as shownby the red line: $v_R = \frac{k_R[C_1]}{[C_1]+K_{C1}} + b$, where K_{C1} = 0.6 ± 0.3 mM^{-1}. This apparent K_{C1} is smaller than that of Br^- (K_{Br-}; Supplementary Fig. 2b), which is the counter anion in C_1. Therefore, the role of the C_1 cation cannot be reliably determined here, nor be compared with other xTAB (x ≥6). Error bars are s.d.

2.3. Debye length calculation

Debye length (κ^{-1}) is a measure of a charge carrier's net electrostatic effect in a solution. At room temperature (20 °C), Debye length (in nm) can be obtained as[1]:

$$\kappa^{-1} = \frac{0.304}{\sqrt{I}}$$

where I is the ionic strength expressed in molar (M). For 7 mM phosphate buffer, $I \approx 0.042$ M, so κ^{-1} = 1.5 nm.

3. Additional figures and tables

Supplementary Table 1. Fitting parameters of ligand competition titration curves with 5-nm Au nanoparticles at 20 °C.

Ligand	K_1 (μM^{-1})	h_1	K_2 (μM^{-1})	h_2	K_1^{-1} (nM)	K_2^{-1} (nM)	P (nM)	Q (nM)
C_6	7.0 ± 5.6	0.95 ± 0.26	N/A	N/A	1.4E+02	N/A	N/A	N/A
C_8	8.1 ± 7.2	0.92 ± 0.18	N/A	N/A	1.2E+02	N/A	N/A	N/A
C_{10}	17 ± 8	0.90 ± 0.09	0.011 ± 0.001	1.25 ± 0.04	59	9.1E+04	100 ± 60	(6.1 ± 1.1) e3
C_{12}	139 ± 63	0.83 ± 0.11	0.021 ± 0.002	1.37 ± 0.10	7.2	4.8E+04	20 ± 8	80 ± 15
C_{14}	255 ± 68	0.80 ± 0.07	0.29 ± 0.06	1.47 ± 0.24	3.9	3.4E+03	12 ± 5	3.0 ± 0.3
C_{16}	409 ± 143	0.75 ± 0.04	0.72 ± 0.05	1.74 ± 0.10	2.4	1.4E+03	7.3 ± 0.9	1.7 ± 0.5
C_{18}	765 ± 191	0.65 ± 0.08	0.76 ± 0.06	1.84 ± 0.13	1.3	1.3E+03	27 ± 1	7.1 ± 1

(cont.)

Ligand	ΔG_1 (kJ/mol)	ΔG_2 (kJ/mol)
C_6	−39.1 ± 31.3	N/A
C_8	−39.4 ± 35.0	N/A
C_{10}	−41.2 ± 19.4	−23.1 ± 2.1
C_{12}	−46.5 ± 21.1	−24.7 ± 2.4
C_{14}	−48.0 ± 12.8	−31.2 ± 6.4
C_{16}	−49.1 ± 17.2	−33.4 ± 2.3
C_{18}	−50.7 ± 12.7	−33.5 ± 2.6

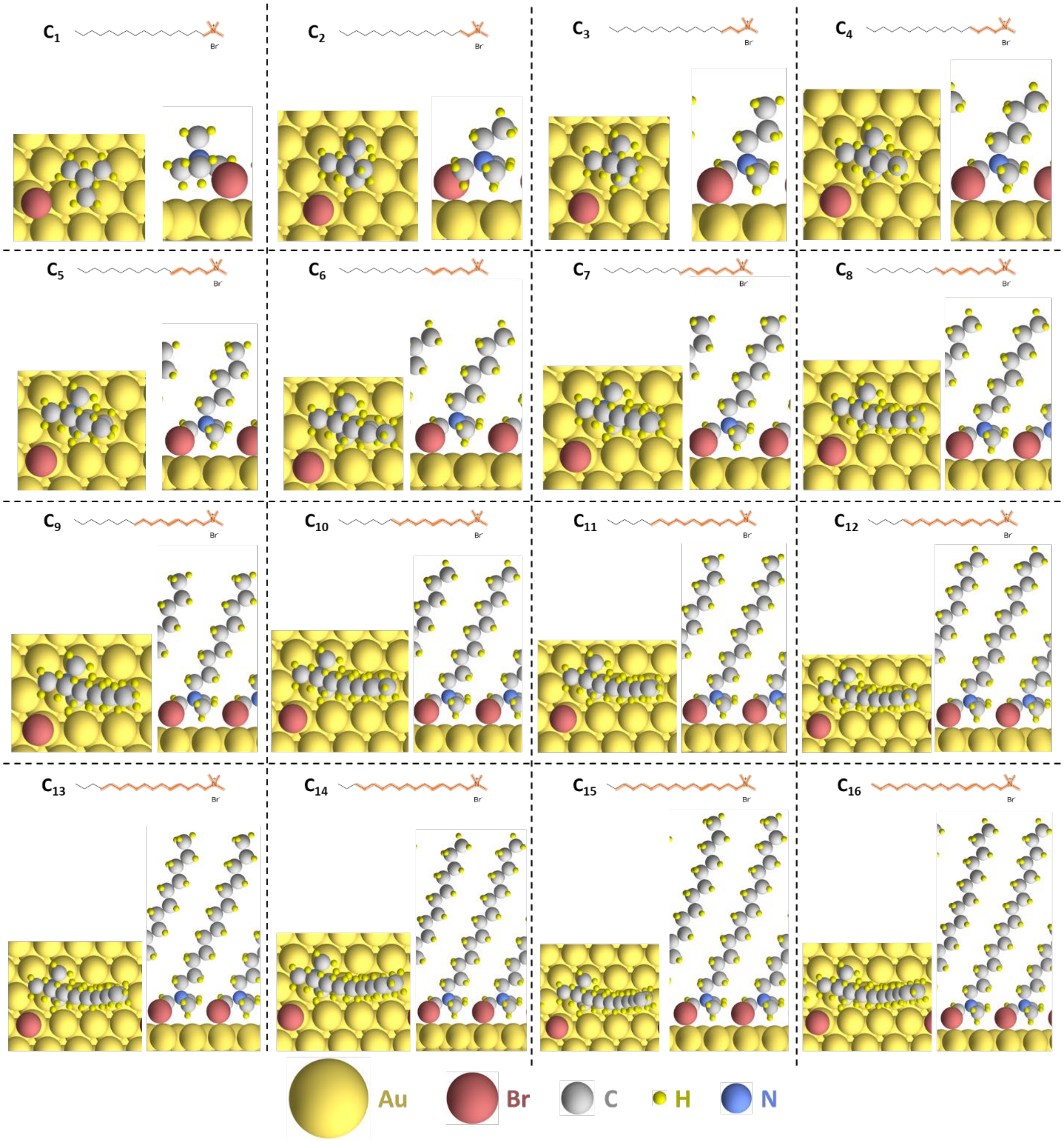

Supplementary Fig. 4. Lowest-energy binding structures of *x*TAB molecules (C_1 – C_{16}) in the standing-up configuration on Au{111} at 1/16 ML coverage. Top (left) and side (right) views for each molecule are shown. The orange shade denotes the actual length of the alkyl chain in each molecule compared to CTAB (C_{16}).

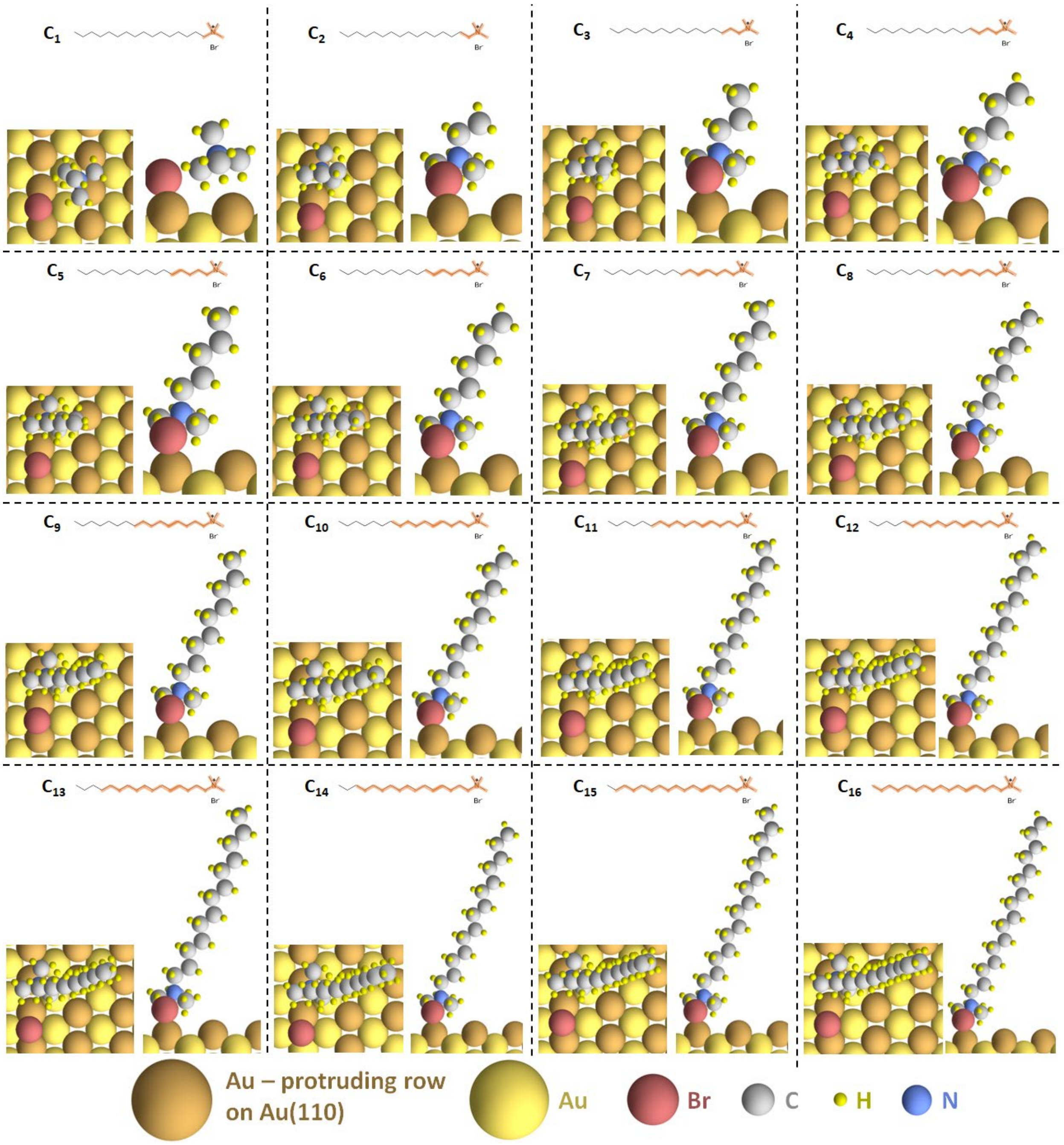

Supplementary Fig. 5. Lowest-energy binding structures of XTAB molecules (C_1 – C_{16}) in the standing-up configuration on Au{110} at 1/16 ML coverage. Top (left) and side (right) views for each molecule are shown. The orange shade denotes the actual length of the alkyl chain in each molecule compared to CTAB (C_{16}).

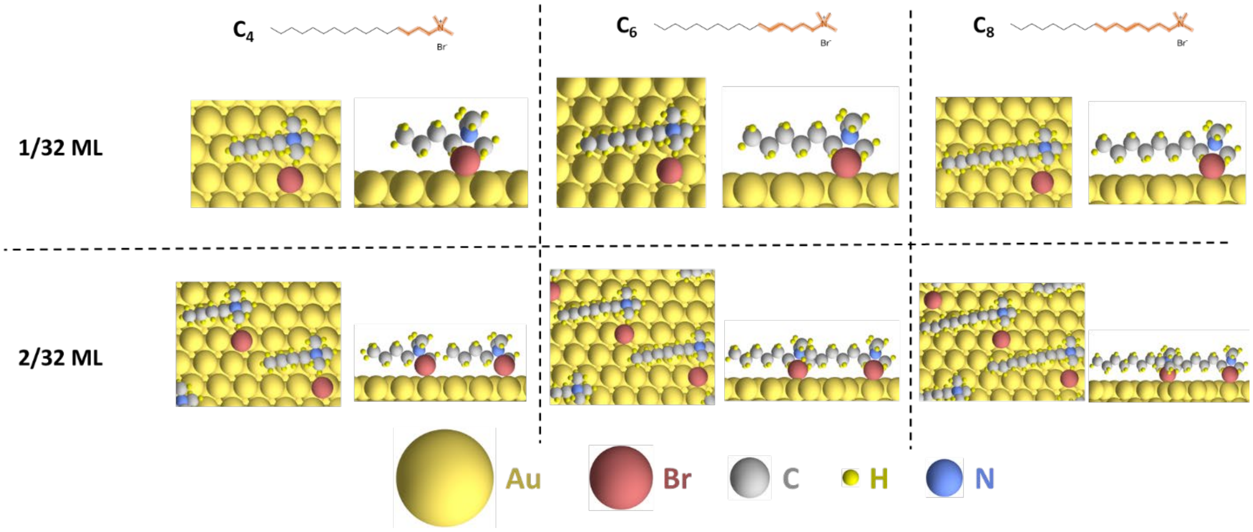

Supplementary Fig. 6. Lowest-energy binding structures of C_4, C_6, and C_8 XTAB molecules in the lying-down configuration on Au{111} at 1/32 ML and 2/32 ML coverages. Top (left) and side (right) views for each molecule are shown. The orange shade denotes the actual length of the alkyl chain in each molecule compared to CTAB (C_{16}).

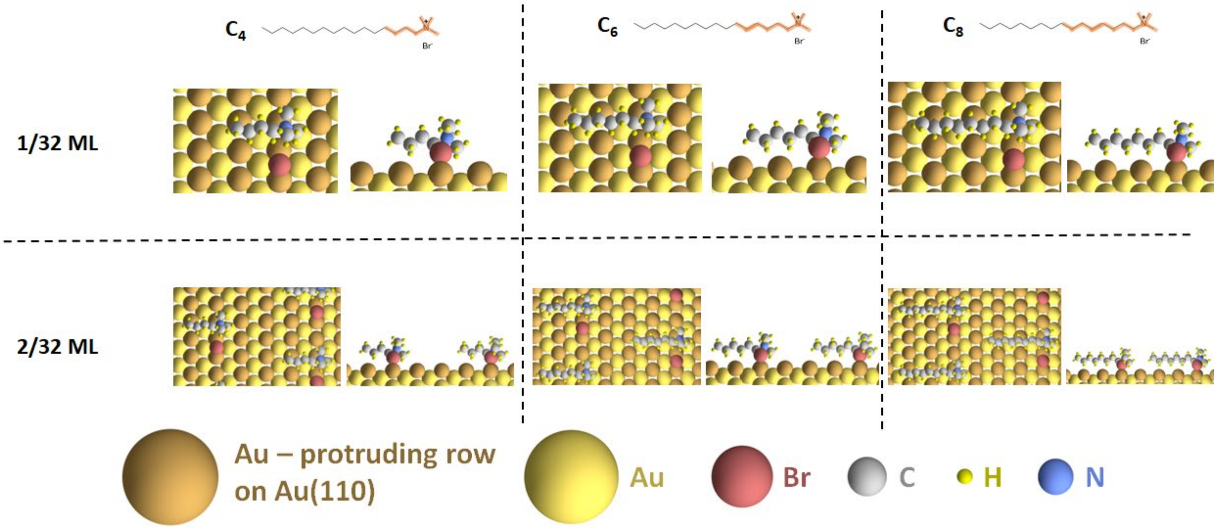

Supplementary Fig. 7. Lowest-energy binding structures of C_4, C_6, and C_8 XTAB molecules in the lying-down configuration on Au{110} at 1/32 ML and 2/32 ML coverages. Top (left) and side (right) views for each molecule are shown. The orange shade denotes the actual length of the alkyl chain in each molecule compared to CTAB (C_{16}).

4. References

1. Israelachvili, J. *Intermolecular and Surface Forces*, 1985. Academic Press.
